# Etomidate-induced hypokalemia in electronic cigarette users: two case reports and literature review

**DOI:** 10.3389/fendo.2024.1321610

**Published:** 2024-05-30

**Authors:** Wenchang Wu, Can Xia, Lulu Gan, Shibo Liao, Yimin Yan

**Affiliations:** ^1^Department of Endocrinology, Xiaogan Hospital Affiliated with Wuhan University of Science and Technology, The Central Hospital of Xiaogan, Xiaogan, Hubei, China; ^2^Medical College, Wuhan University of Science and Technology, Wuhan, China

**Keywords:** etomidate, hypokalemia, adrenal hyperplasia, 11-beta-hydroxylase, hypertension

## Abstract

Hypokalemia is a common clinical condition that can lead to muscle weakness, difficulty breathing, malignant arrhythmias, and even death. This report describes two cases of severe hypokalemia resulting from the use of electronic cigarettes containing etomidate, both accompanied by varying degrees of adrenal hyperplasia. In both cases, the patients were admitted to the hospital with lower limb weakness and difficulty walking. Relevant examinations revealed low blood potassium, low cortisol, high adrenocorticotropic hormone, low renin, and low aldosterone levels in the patients, with Case 2 also having significant hypertension. In both cases, adrenal CT scans showed thickening of the adrenal glands. After the delivery of potassium supplementation in both cases, blood potassium levels gradually returned to normal and muscle strength gradually improved. The case reports are followed by a review of the literature on etomidate and its related mechanisms of action with discussion of its association with hypokalemia.

## Introduction

Etomidate is a non-barbiturate, sedative-hypnotic drug used to induce anesthesia. Its primary mechanism of action is positive allosteric modulation of the γ-aminobutyric acid type A receptor, which enhances the inhibitory effects of the neurotransmitter γ-aminobutyric acid (1). The advantages of etomidate include rapid onset of effects, short duration of effects, fast drug metabolism, no histamine release, and stable hemodynamics.

Numerous studies have shown that etomidate can cause adrenal insufficiency primarily by inhibiting 11β-hydroxylase, and this effect is dose-dependent. However, very few studies on the relationship between etomidate and hypokalemia have been reported. Recently, during our clinical work, we admitted two patients with hypokalemia caused by the use of electronic cigarettes containing etomidate. Here we present these two cases and review the relevant literature to better understand the association between etomidate and hypokalemia.

## Cases Report

### Case 1

A 29-year-old Han male was admitted to the hospital presenting with “lower limb weakness for 2 days”. The patient had experienced sudden onset of lower limb weakness 2 days previously that made him unable to walk and was accompanied by abnormal sensations such as pain and numbness in the lower limbs. His blood potassium level was measured to be 1.69 mmol/L in the outpatient department, leading to the diagnosis of hypokalemia and hospitalization.

Physical examination: body temperature, 36.3°C; pulse rate, 90 beats/min; respiratory rate, 19 breaths/min; blood pressure, 132/88 mmHg; and body mass index (BMI), 27.4 kg/m^2^. No significant abnormalities were found on cardiovascular, pulmonary, or abdominal examinations. Muscle strength was grade 4 for all limbs, with moderate muscle tone. The patient stated he had no significant medical history. He reported intermittent use of electronic cigarettes containing etomidate for the previous 3 months. Each e-cigarette used by the patient contains 2.0 ml of e-liquid. The patient typically consumes one e-cigarette every two days. The patient’s parents have no history of smoking and exhibit no symptoms indicative of hypokalemia.

**Figure 1:**
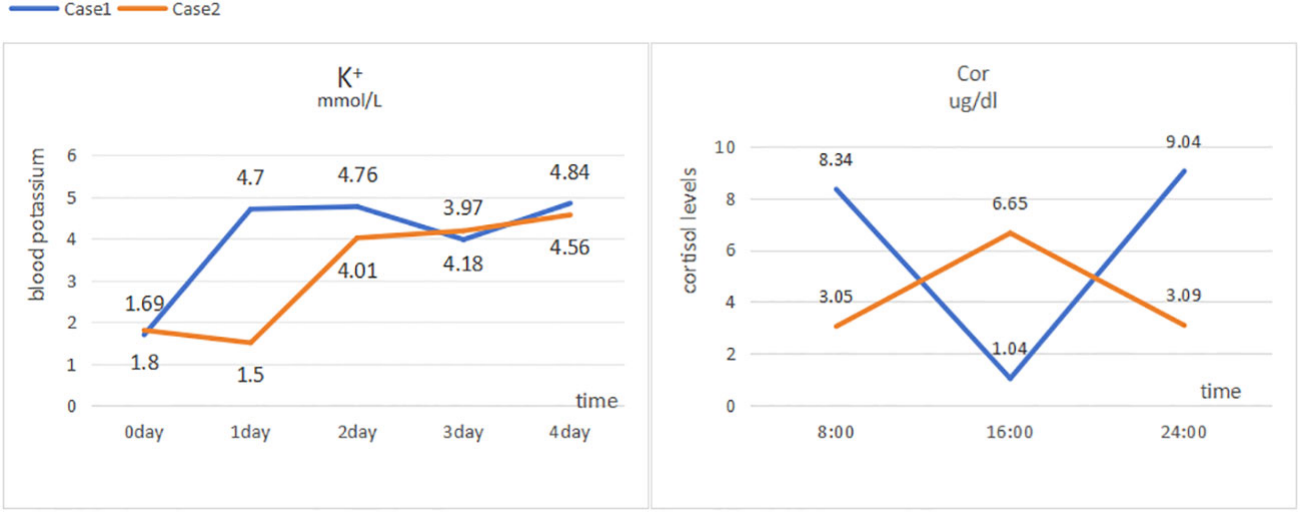
Fluctuations in blood potassium and cortisol levels in Case 1 and Case 2.

**Figure 2:**
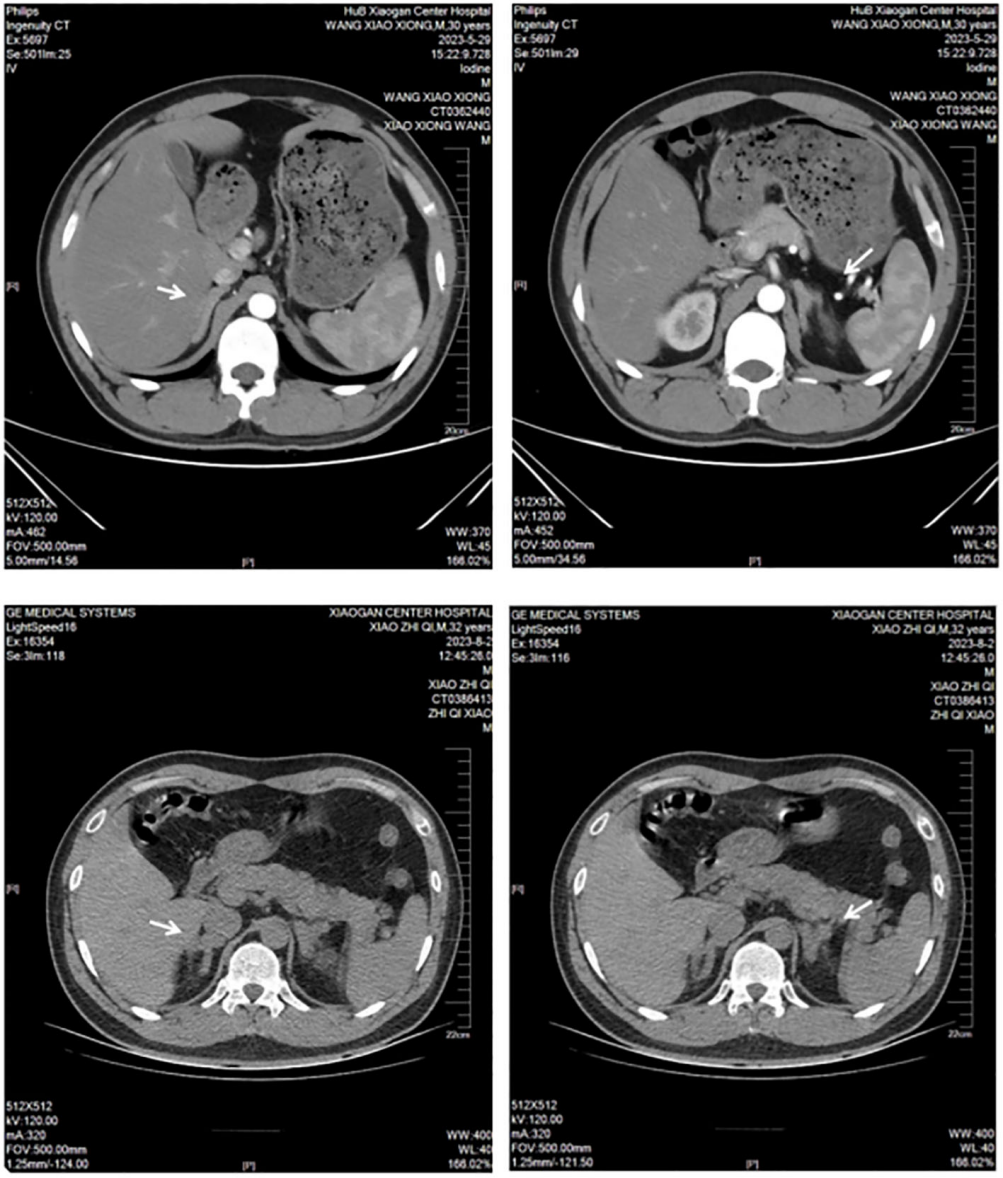
Adrenal CT images for Case 1 and Case 2.

Laboratory tests: blood potassium, 4.7 mmol/L; 24-h urinary potassium excretion, 123.31 mmol/24 h (reference range: 25–100 mmol/24 h); 24-h urine volume, 1900 mL; cortisol levels: 8:00 AM 8.34 µg/dL (reference range: 6.2–19.4 µg/dL), 4:00 PM 1.04 µg/dL (reference range: 2.3–11.9 µg/dL), 12:00 AM 9.04 µg/dL (**Figure 1**); adrenocorticotropic hormone (ACTH), 52 pg/mL (reference range: 6–48 pg/mL); aldosterone in supine position, 5.01 pg/mL (reference range: 60–360 pg/mL); renin activity in supine position, 0.56 ng/mL/h (reference range: 0.15–2.33 ng/mL/h); aldosterone in upright position, 5.000 pg/mL (reference range: 50–313 pg/mL); and renin activity in upright position, 0.64 ng/mL/h (reference range: 1.31–3.95 ng/mL/h). Endocrine examination revealed: prolactin, 16.60 ng/mL (reference range: 2.55–16.04 ng/mL); follicle-stimulating hormone, 1.75 mIU/mL (reference range: 1.5–11.8 mIU/mL); luteinizing hormone, 3.63 mIU/mL (reference range: 1.1–25 mIU/mL), estradiol, 56.40 pg/mL (reference range: 7.63–42.6 pg/mL); progesterone, 0.41 ng/mL (reference range: 0.05–0.149 ng/mL); testosterone, 0.89 ng/mL (reference range: 2.2–10.5 ng/mL); and parathyroid hormone, 63.94 pg/mL (reference range: 15–65 pg/mL). Adrenal CT scan showed bilateral adrenal gland thickening (**Figure 2**). No significant abnormalities were detected on routine blood tests, blood gas analysis, glucose tolerance test, thyroid function tests, catecholamine measurement, and antinuclear antibody spectrum analysis. Thyroid ultrasound, cardiac ultrasound, and chest CT were normal.

At admission, the patient’s blood potassium level was 1.69 mmol/L, and he was given intravenous infusion of 10% potassium chloride solution and oral administration of 20 mL of 10% potassium chloride solution. His blood potassium level did not return to normal readily, and an urgent blood gas analysis showed a potassium ion level of 1.5 mmol/L and an actual bicarbonate level of 27.5 mmol/L. The patient was then treated with central venous infusion of 9 g potassium chloride at a rate of 0.5 g/h, and his blood potassium level had risen to 4.01 mmol/L when rechecked. His muscle weakness gradually disappeared, and he was prescribed oral sustained-release potassium chloride tablets (0.5 g three times a day). With this treatment, his blood potassium level remained within the normal range. Before discharge, his blood potassium level was measured to be 4.84 mmol/L, with an ACTH level of 5 pg/mL and 24-h urinary potassium excretion of 31.60 mmol. At that time, the following cortisol levels were measured: 8:00 AM 1.02 µg/dL (reference range: 6.2–19.4 µg/dL), 4:00 PM 2.23 µg/dL (reference range: 2.3–11.9 µg/dL), and 12:00 AM 14.01 µg/dL.

### Case 2

A 32-year-old Han male patient was admitted to the hospital for “weakness in all limbs for half a day.” The patient experienced sudden onset of weakness in all limbs without an obvious cause approximately 12 hours previously, accompanied by difficulties in standing up, turning over, and walking. His blood potassium level was measured to be 1.80 mmol/L in the outpatient department, leading to the diagnosis of hypokalemia and hospitalization.

Physical examination: body temperature, 36.5°C; pulse rate, 86 beats/min; respiratory rate, 19 breaths/min; blood pressure, 161/96 mmHg; and BMI, 24.45 kg/m^2^. No significant abnormalities were found on cardiovascular, pulmonary, or abdominal examinations. Muscle strength was grade 2 for all limbs, with moderate muscle tone. The patient reported having no significant medical history and using electronic cigarettes containing etomidate for more than 2 months. Similarly, the patient’s parents lack a history of smoking and display no symptoms indicative of hypokalemia.

Laboratory tests: blood potassium, 1.5 mmol/L (reference range: 3.5–5.3 mmol/L); 24-h urinary potassium excretion, 53.58 mmol/24 h (reference range: 25–100 mmol/24 h), 24-h urine volume, 2820 mL; cortisol levels: 8:00 AM 3.05 µg/dL (reference range: 6.2–19.4 µg/dL), 4:00 PM 6.65 µg/dL (reference range: 2.3–11.9 µg/dL), 12:00 AM 3.09 µg/dL (**Figure 1**); ACTH, 94 pg/mL (reference range: 6–48 pg/mL); aldosterone in supine position, 85.4 pg/mL (reference range: 60–360 pg/mL); renin activity in supine position, 0.38 ng/mL/h (reference range: 0.15–2.33 ng/mL/h); aldosterone in upright position, 63.39 pg/mL (reference range: 50–313 pg/mL); and renin activity in upright position, 0.38 ng/mL/h (reference range: 1.31–3.95 ng/mL/h). Endocrine examination revealed: estradiol, 48.60 pg/mL (reference range: 7.63–42.6 pg/mL); progesterone, 0.95 ng/mL (reference range: 0.05–0.149 ng/mL); and growth hormone, 138.40 pg/mL (reference range: 200–8000 pg/mL). Adrenal CT scan showed bilateral adrenal gland thickening (**Figure 2**). Routine blood tests, liver and kidney function tests, glucose tolerance test, thyroid function testing, and coagulation function testing showed no significant abnormalities. Thyroid ultrasound, cardiac ultrasound, and chest CT were normal.

At admission, his blood potassium level was 1.80 mmol/L. The patient immediately received intravenous infusion of 10% potassium chloride solution and oral administration of 20 mL of 10% potassium chloride solution. His blood potassium level did not return to normal readily, and urgent blood gas analysis showed a potassium ion level of 1.5 mmol/L and an actual bicarbonate level of 27.5 mmol/L. The patient was then treated with a central venous infusion of 9 g potassium chloride at a rate of 0.5 g/h. When rechecked, the patient’s blood potassium level was 4.01 mmol/L, and muscle weakness gradually disappeared. The patient was later switched to oral sustained-release potassium chloride tablets (0.5 g), and his blood potassium level was maintained within the normal range. Before discharge, the patient had a blood potassium level of 4.56 mmol/L, an ACTH level of 53 pg/mL, and a 24-h urinary potassium excretion measurement of 53.58 mmol. His cortisol levels were as follows: 8:00 AM 10.47 µg/dL (reference range: 6.2–19.4 µg/dL), 4:00 PM 4.20 µg/dL (reference range: 2.3–11.9 µg/dL), and 12:00 AM 1.88 µg/dL.

In both reported cases, the patients presented with hypertension, hypokalemia, high ACTH, low cortisol, low renin, low aldosterone, and bilateral adrenal gland thickening (**Table 1**). Both patients also had a history of using electronic cigarettes containing etomidate. The patients were advised to quit smoking after discharge and to continue taking oral sustained-release potassium chloride tablets (0.5 g three times a day). Follow-up through telephone consultation confirmed no recurrence of hypokalemia symptoms after discharge.

**Table 1:** Laboratory test results for Case 1 and Case 2.

Laboratory test	Case 1		Case 2		Reference range
	Hospitalized	Discharge	Hospitalized	Discharge	
Potassium ion, mmol/L	1.69	4.84	1.80	4.56	3.5–5.3
Cortisol, µg/dL					
8 AM	8.34	1.02	3.05	10.47	6.2–19.4
4 PM	1.04	2.23	6.65	4.20	2.3–11.9
12 AM	9.04	14.01	3.09	1.88	
Corticotrophin, pg/mL	52	5	94	53	6–48
Aldosterone, pg/mL					
Standing	5.00		63.39		50–313
Supine	5.01		85.40		60–360
Plasma renin activity, ng/ml/h					
Standing	0.64		0.38		1.31–3.95
Supine	0.56		0.38		0.15–2.33

## Discussion

The use of etomidate as a γ-aminobutyric acid type A receptor agonist to induce anesthesia has the advantage of a minimal impact on the cardiovascular system. Etomidate typically does not induce significant hypotension during anesthesia induction at a dosage of 0.3 mg/kg. This stability is due to etomidate’s minimal effect on sympathetic tone and its ability to preserve autonomic reflexes, including the baroreflex (2). The drug achieves this by acting as an agonist at α2-adrenoceptors, particularly the α2B subtype, which plays a key role in mediating peripheral vasoconstriction (3). Multiple studies have demonstrated that doses of etomidate used for anesthesia result in minimal changes in heart rate (less than 10%), while maintaining stable hemodynamic parameters such as central venous pressure, pulmonary artery pressure, cardiac index, and systemic vascular resistance (4). This favorable cardiovascular profile makes etomidate an optimal choice for induction of anesthesia in patients who are hemodynamically unstable or suffer from cardiac conditions. Significant adverse reactions to etomidate include postoperative nausea, vomiting, pain upon injection, muscle spasms, and involuntary muscle movements. It has been proposed that hemolysis could be a side effect associated with the presence of propylene glycol in etomidate formulations. In order to minimize these adverse effects, the World Health Organization (WHO) advises that the daily consumption of propylene glycol should not exceed 25 mg/kg (5).

Additionally, the discovery of adrenal toxicity has limited the widespread clinical use of etomidate, and it is currently only used for induction of transient anesthesia in hemodynamically unstable patients. Because etomidate is not classified as a controlled psychotropic or anesthetic drug, a certain risk of abuse exists. Recently, electronic cigarettes containing etomidate have emerged, and adverse reactions to these products have gradually gained widespread attention. Here we report the cases of two patients who after smoking electronic cigarettes containing “etomidate,” experienced severe hypokalemia with varying degrees of muscle weakness and even respiratory distress. Although the patients’ symptoms eventually resolved with potassium supplementation, the impact of these events should be considered.

**Figure 3:**
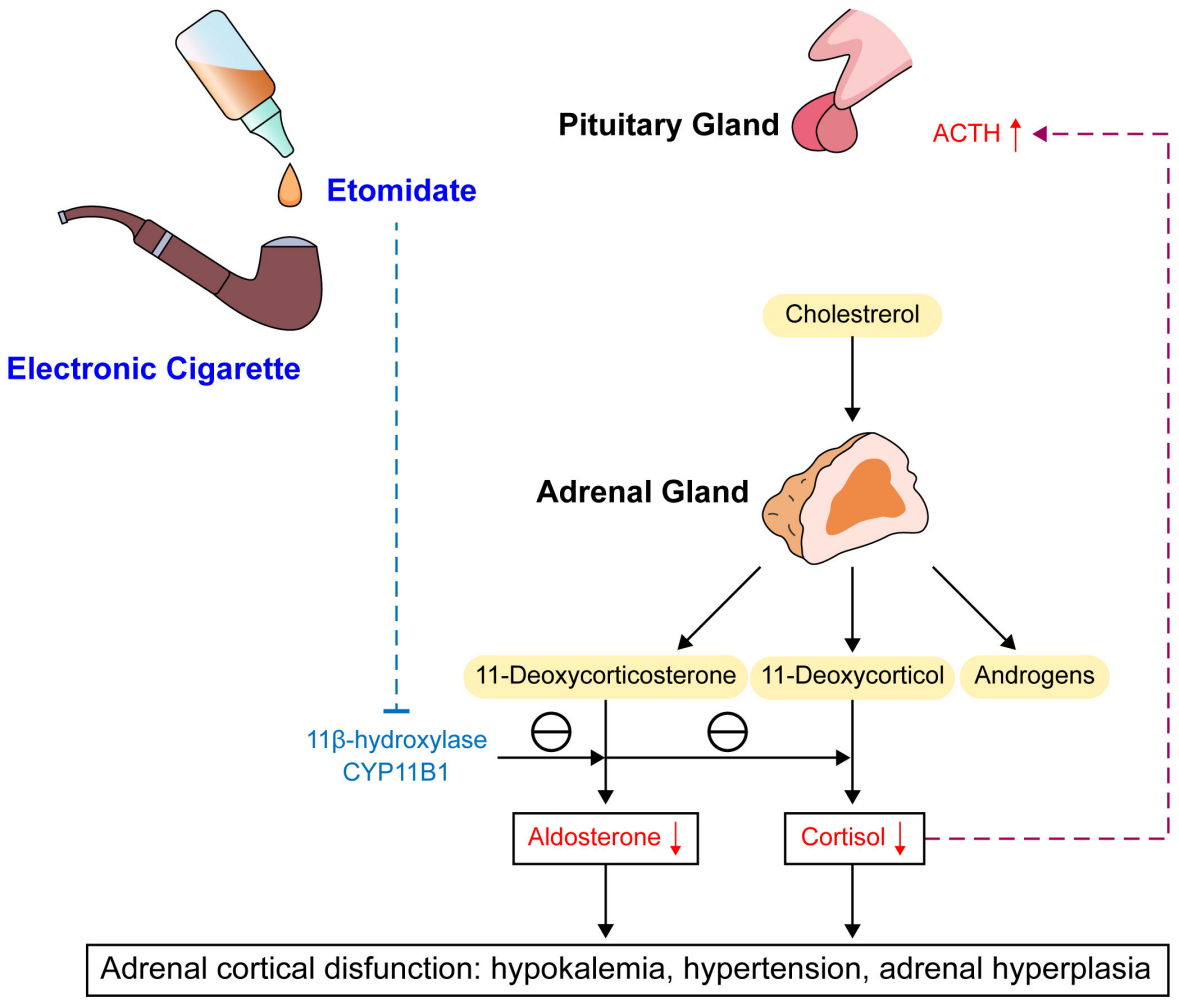
Mechanism and consequences of etomidate. ACTH, adrenocorticotrophic hormone; CYP11B1, Cytochrome p450 enzyme 11β-hydroxylas.

A significant association of etomidate with adrenal insufficiency has been established. Etomidate specifically and reversibly blocks 11α-hydroxylation and 11β-hydroxylation in adrenal steroid synthesis, leading to decreases in the synthesis of cortisol, corticosterone, and aldosterone (6) (**Figure 3**). Cortisol is the most abundant endogenous glucocorticoid (7). Recent research demonstrated that within a certain dosage range, single dosing and/or continuous infusion of etomidate can reduce plasma cortisol levels, although the levels remain within the normal physiological range (8). Pre-administration of vitamin C before etomidate injection mitigates adrenal suppression and diminishes the extent of serum cortisol reduction attributed to etomidate. Beyond its antioxidant properties, vitamin C additionally counteracts the suppressive impact of etomidate on adrenal gland functionality (9).

Additionally, adrenal cortex responsiveness to adrenocorticotropic hormone decreases. The reduction in cortisol level can provide feedback to stimulate the release of adrenocorticotropic hormone, causing adrenal hypertrophy and promoting synthesis of cortisol and corticosterone precursors. Through its effects on the activity of 11β-hydroxylase and 17α-hydroxylase, which result in decreased cortisol synthesis from 11-deoxycortisol and a decrease in aldosterone, etomidate can lead to temporary adrenal insufficiency. Notably, this effect of inhibiting adrenal cortex enzyme 11β-hydroxylase makes etomidate an effective acute treatment for severe Cushing’s syndrome. In these patients, etomidate takes effect quickly and is the only steroidogenesis enzyme inhibitor that can be delivered parenterally, lowering the risk of liver damage compared to other drug treatments, such as ketoconazole (10).

Studies of the specific molecular mechanism of its effects indicate that etomidate, as an imidazole derivative, can inhibit cytochrome P450 enzyme 11β-hydroxylase (CYP11B1) in adrenal cortex cells, with the imidazole ring possibly being the main determinant of etomidate binding to CYP11B1. CYP11B1 is a key enzyme in glucocorticoid synthesis necessary for the conversion of 11-deoxycortisol to cortisol and 11-deoxycorticosterone to corticosterone (11). Animal experiments in crab-eating macaques showed that ketoconazole also lowers cortisol and corticosterone levels by inhibiting CYP11B1, with disproportionally greater increases in 11-deoxycortisol and 11-deoxycorticosterone compared with the decreases in cortisol and corticosterone. Long-term use of ketoconazole also was shown to increase pregnenolone and androstenedione production, possibly due to compensatory reactions to the decrease in cortisol (12). Etomidate and ketoconazole are both imidazole derivatives, which also explains the hormonal abnormalities observed in the present cases. Inhibition of CYP11B1 can increase the concentrations of precursor substances such as 11-deoxycorticosterone, 11-deoxycortisol, progesterone, and 17-hydroxyprogesterone (13). 11-Deoxycorticosterone is a precursor to aldosterone and has approximately 1/20 to 1/30 the mineralocorticoid activity, and an increase in 11-deoxycorticosterone can have effects such as sodium and water retention and increased potassium excretion. A study on adrenal functional tumors caused by non-aldosterone–dependent mineralocorticoid secretion found that such adrenal lesions can produce a large amount of 11-deoxycorticosterone, and the clinical manifestations are somewhat similar to those of primary aldosteronism, usually including hypertension and moderate to severe hypokalemia (14).

Regarding the timing of the effects of etomidate, a single dose of etomidate given before surgery usually results in a return to baseline levels of the inhibited enzymes by 24 hours after anesthesia induction, while continuous use leads to an inhibitory effect lasting longer than 24 hours, with possibly dose-dependency (15). Compared with propofol and dexmedetomidine, etomidate has a stronger adrenal suppression effect, and adrenal insufficiency can persist for a long time after discontinuation of continuous etomidate administration (16). Adrenal cortex dysfunction caused by inhibition of 11β-hydroxylase can manifest as low cortisol, high ACTH, low or normal aldosterone, high sex hormones, moderate to severe hypokalemia, hypertension, and compensatory adrenal hyperplasia. This is consistent with the clinical characteristics observed in the two reported cases after smoking of electronic cigarettes containing “etomidate.” Considering the known persistence of the inhibitory effect of etomidate with continuous use as well as the significant dose-dependency, long-term smoking of electronic cigarettes containing “etomidate” likely prolongs the inhibitory effect of the drug, allowing the development of severe hypokalemia, adrenal crisis, and a significantly increased risk of death. Thus, greater awareness of the risks of unregulated use of products containing etomidate is needed. For conditions involving cortisol excess though, the good therapeutic effects of etomidate warrant further investigation of its clinical value (17).

## Conclusion

Our analysis of the two case reports revealed that both patients had been using electronic cigarettes containing etomidate for several months. Following this exposure, they presented with clinical and laboratory signs of hypokalemia, hypertension, elevated ACTH levels, and reduced renin and aldosterone levels. Imaging studies indicated adrenal gland thickening. Upon discharge and subsequent follow-up, it was noted that cessation of e-cigarette use led to the resolution of symptoms, including fatigue, abnormal sensations, and hypokalemia. This report describes the diagnosis and treatment processes for two cases of etomidate-related hypokalemia in adult male patients with a recent history of smoking electronic cigarettes containing “etomidate.” The mechanisms of etomidate action were reviewed to better understand the development of etomidate-induced hypokalemia and adrenal insufficiency. Based on the presented cases and reviewed mechanistic information, healthcare providers should be aware of the possibility of that hypokalemia or symptoms of adrenal hyperplasia may be related to electronic cigarette use. Patients who present with these conditions and have a history of smoking should be asked about electronic cigarette use, and close monitoring of their blood potassium levels along with relevant examinations are needed to facilitate early treatment and improve their prognosis.

## Author contributions

WW: Writing – review & editing. CX: Writing – review & editing. LG: Writing – review & editing. SL: Writing – review & editing. YY: Writing – review & editing.
